# Effects on hemodynamic enhancement and discomfort of a new textile electrode integrated in a sock during calf neuromuscular electrical stimulation

**DOI:** 10.1007/s00421-023-05212-5

**Published:** 2023-05-05

**Authors:** C. Sundström, R. Juthberg, J. Flodin, L. Guo, N.-K. Persson, P. W. Ackermann

**Affiliations:** 1 Department of Molecular Medicine and Surgery, Karolinska Institutet, Stockholm, Sweden; 2 Polymeric E- Textiles and Smart Textiles University of Borås, Borås, Sweden; 3 Department of Trauma, Acute Surgery and Orthopaedics, Karolinska University Hospital, 171 76 Stockholm, Sweden

**Keywords:** Electric stimulation therapy, Textile electrodes, Motor point, NMES, Hemodynamics, Pain

## Abstract

**Purpose:**

To compare fixed transverse textile electrodes (TTE) knitted into a sock versus motor point placed standard gel electrodes (MPE) on peak venous velocity (PVV) and discomfort, during calf neuromuscular electrical stimulation (calf-NMES).

**Methods:**

Ten healthy participants received calf-NMES with increasing intensity until plantar flexion (measurement level I = ML I), and an additional mean 4 mA intensity (ML II), utilizing TTE and MPE. PVV was measured with Doppler ultrasound in the popliteal and femoral veins at baseline, ML I and II. Discomfort was assessed with a numerical rating scale (NRS, 0–10). Significance was set to *p* < 0.05.

**Results:**

TTE and MPE both induced significant increases in PVV from baseline to ML I and significantly higher increases to ML II, in both the popliteal and femoral veins (all *p* < 0.001). The popliteal increases of PVV from baseline to both ML I and II were significantly higher with TTE versus MPE (*p* < 0.05). The femoral increases of PVV from baseline to both ML I and II were not significantly different between TTE and MPE. TTE versus MPE resulted at ML I in higher mA and NRS (*p* < 0.001), and at ML II in higher mA (*p* = 0.005) while NRS was not significantly different.

**Conclusion:**

TTE integrated in a sock produces intensity-dependent increases of popliteal and femoral hemodynamics comparable to MPE, but results in more discomfort at plantar flexion due to higher current required. TTE exhibits in the popliteal vein higher increases of PVV compared to MPE.

**Trial registration:**

Trial_ID: ISRCTN49260430. Date: 11/01/2022. Retrospectively registered.

## Introduction

Venous stasis is one of the major causes of venous thromboembolism (VTE), which often starts as a deep vein thrombosis (DVT). VTE-prevention targeting venous stasis of the calf include passive compression socks and active mechanical interventions, such as intermittent pneumatic compression and neuromuscular electrical stimulation (NMES), of which the latter today have limited effect due to poor patient compliance (Hajibandeh et al. 2015). Calf-NMES could potentially be integrated into socks resulting in better mobility and thereby improved patient compliance.

Previous studies have demonstrated that textile electrodes are well suited for NMES (Gniotek et al. 2011; Crema et al. 2018). However, as textile surfaces are rather complex, the interface of textile electrodes and the skin behaves differently than for example for standard gel electrodes. This means that findings for other electrode types cannot directly be translated and assumed to be the same for textile electrodes (Euler et al. 2021a, b). Furthermore, there are to the best of our knowledge no prior studies that have investigated the venous hemodynamic effects of NMES via textile electrodes integrated in fixed positions in a garment, compared to NMES via standard gel electrodes placed on individual motor points (MP).

To address the above issues, we designed a novel NMES sock with textile electrodes and tested its efficacy. We believe that such novel NMES sock in the future may have the potential to produce intensity-dependent increases in peak venous velocity (PVV) of both the popliteal and femoral veins, which have been associated with an efficient VTE-prevention (Hajibandeh et al. 2015; Williams et al. 2017). This is important since NMES via textile electrodes must be shown to produce comparable PVV as compared to NMES via standard gel electrodes, to become a viable alternative if used for VTE-prevention. To have potential for VTE-prevention in a clinical setting the PVV needs to increase in both the popliteal and femoral vein since the main location of blood clotting is in the deep veins of the calf, however, a large proportion of blood clots also start more proximally such as after trauma, surgery and immobilization.

When using standard gel electrodes, a *motor point electrode* (MPE) setup has demonstrated reduced discomfort compared to random electrode placement (Lyons et al. 2004; Gobbo et al. 2011, 2014). Whether textile electrodes in fixed positions according to a MP map (Botter et al. 2011), i.e., a *transverse textile electrode* (TTE) setup, demonstrate non-inferior comfort compared to traditional MPE on the calf has not been investigated.

In this efficacy study, we hypothesized that TTE would result in no difference in effect on hemodynamics and comfort compared to MPE. Thus, the primary aim of this study was to examine whether calf-NMES, using one new TTE-setup with textile electrodes in one-size fits all sock and one established MPE-setup, could both induce significant intensity-dependent increases of PVV in the popliteal and femoral veins related to the current intensity applied. The secondary aim of this study was to explore whether TTE could produce no difference in effect on hemodynamics and comfort compared to the established MPE.

## Materials and methods

### Participants

Ten healthy individuals (five men and five women) were included in the study. All participants completed a questionnaire before entering the study (Table 1), including measurements to calculate body composition using the US Navy formula (Shaheen et al. 2019) and an estimation of their physical activity on a scale of 1–6 using the Grimby/Frändin activity scale stated as PAS (Grimby et al. 2018), 1 represents no physical activity and 6 intense work out several times a week. Participants aged between 18 and 75 years were eligible for inclusion. The exclusion criteria were pregnancy, skin ulcers, previous surgery on blood vessels of the lower limbs, pacemaker, intracardiac defibrillator, advanced heart disease, kidney failure, cancer and neuromuscular or metabolic disease. The study included a MP scan and sessions where Doppler ultrasound was performed during NMES stimulation to measure PVV, all while the participant was placed in a semirecumbent position.

**Table 1:** Characteristics of the participants (*n* = 10)

Variable	Median (Range)
Age (years)	27 (24–55)
Height (cm)	174 (164–187)
Weight (kg)	64 (56–110)
BMI (kg/m^2^)	22 (19–32)
Body fat (%)	20 (9–30)
Waist circumference (cm)	73 (67–114)
PAS, range 1–6	5 (3–6)

*M*  median, *R*  Range, *BMI*  body mass index, *PAS*  Physical activity scale

### Electrode-setups

In this study, two different electrode setups for applying NMES to the calf were tested and compared regarding various outcomes. The electrode setups differed regarding electrode- type, and placement and were compared to investigate if textile electrodes knitted in fixed transverse positions in a sock, would yield a non-inferior increase of PVV from baseline compared to standard gel electrodes placed at individual MP.

**Fig. 1:**
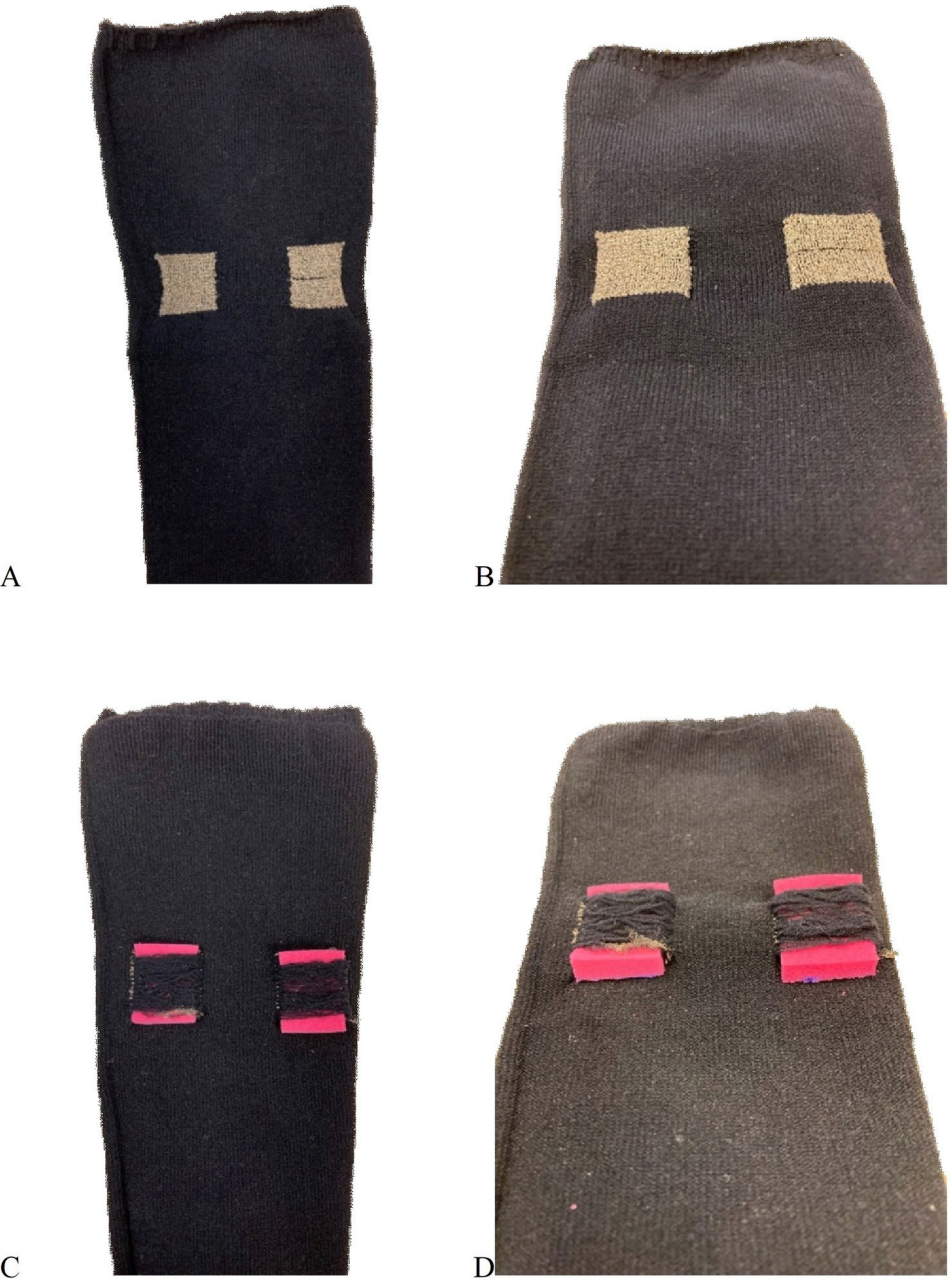
Displaying the transverse textile electrodes. Sock with knitted transverse textile electrodes, the face side (**a**–**b**), the back side with pink melamine sponges attached (**c**–**d**) and the sock displayed in an oblique view (**b**, **d**)

**Fig. 2:**
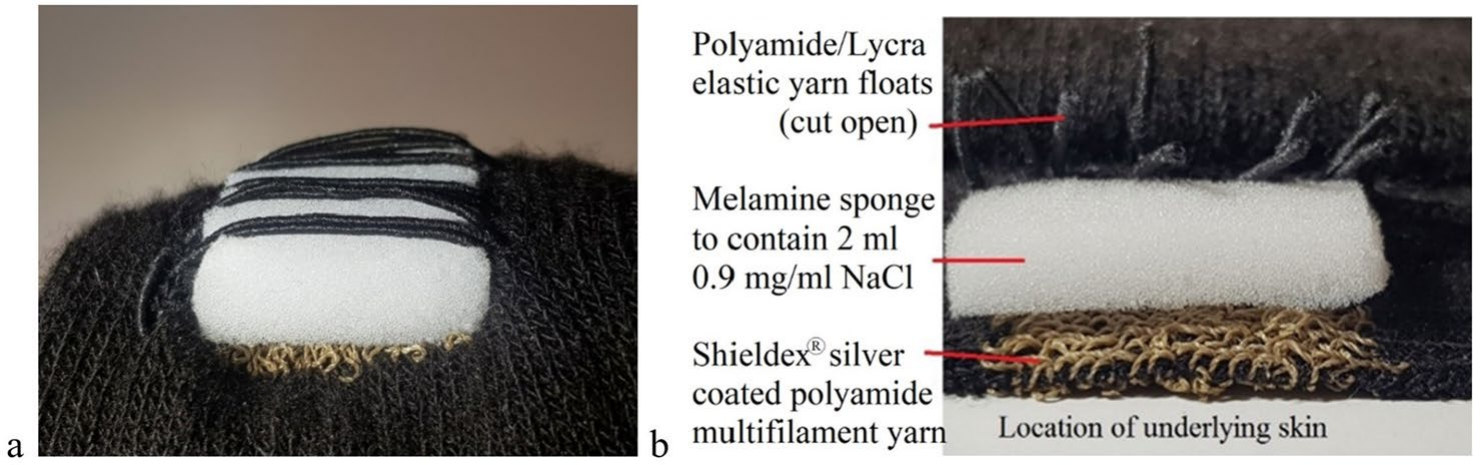
The layers of the transverse textile electrodes. **a** A longitudinal view of an uncut textile electrode, and **b** a transversal view of a longitudinal cut through section displaying the layers of the textile electrode. For better visibility of the layers, some of the floats have been removed in Fig. 2**a**–**b**, as compared to Fig. 1c–d

The first electrode setup designated TTE, short for *transverse textile electrode* setup, consisted of two rectangular textile electrodes (2 × 2.5 cm) transversally knitted with the inner edges 1.8 cm apart into the back of a one-size-fits-all sock (polyamide/Lycra blended yarn) (Fig. 1a–b). The electrodes were placed approximately at the largest circumference of the calf with the electrodes positioned to cover areas of the calf, given the large interindividual variation of MP locations, which according to a MP map created by Botter et al. 2011 in general has a high likelihood of containing a MP (Botter et al. 2011). When the sock was applied to the calf the textile electrodes stretched to the size of approximately 3 × 3 cm, and the distance between the inner edges of the textile electrodes increased to approximately 3 cm. The same socks were used for all the subjects; therefore, the electrode size might alter slightly due to the stretch of the textile as well as the localization of the electrodes may change slightly. The electrodes were knitted into the sock using industrial intarsia knitting which allows for seamless integration of patterns of functional components in a single process. The material of the electrodes was silver coated polyamide multifilament yarn, with trade name Shieldex® (produced by Statex Produktions und Vertriebs GmbH). On the outside of the sock, the electrodes medial and lateral edges were covered with elastic yarn floats (polyamide/Lycra). The purpose of the floats was to hold a moisture-containing sponge (0.5 × 3 × 3 cm, injected with 2 ml NaCl 0.9 mg/ml) in direct contact with each of the underlying electrodes, to increase the local pressure and humidity of the electrode/skin-interface, and thus uphold an adequate electrical conduction (Fig. 2) (Euler et al. 2021a, b). Since the TTE-sock was only a prototype, a simple “off the shelf” melamine cleaning sponge was used as the moisture container. The different layers of the textile electrode are displayed in Fig. 2. Crocodile clips were used to connect the wires from the NMES device to the outside of the electrode fabric of the TTE.

The second electrode setup utilized commercially available standard gel electrodes (Compex Snap, Performance, DJO Global, USA, 5 × 5 cm) manually trimmed to squares sized 3 × 3 cm, to match the size of the TTE. Each standard gel electrode had a snap-on button to which each wire from the NMES-device was attached. The standard electrodes were placed on the skin areas of the calf, one on the medial side and one the lateral side, that required the least NMES current intensity to trigger a calf muscle response, i.e., the “best” MP, as determined by a standard motor point scan. This electrode setup was designated MPE, short for *motor point electrode* setup.

### Motor point scan

The best MPs were found by scanning one half of the calf at a time (medial/lateral), using the NMES device’s 3 Hz sinusoidal wave motor scan program (Chattanooga Physio constant current generator, DJO Nordic, Malmoe, Sweden). Prior to the MP scan, the side of the calf about to be scanned was covered by a thin layer of conductive gel. A reference electrode (Compex Snap, Performance, DJO Global, USA, 5 × 5 cm) was placed on the contralateral side from the MP scan over the largest circumference of the calf, on a distance from the calf’s midline corresponding to 15% of the calf’s largest circumference. The definition of a MP was the same as in our previous study (Schriwer et al. 2023) e.g., as a location on the skin that resulted in a muscle twitch at the lowest level of stimulation compared to the closest surrounding area (Moon et al. 2012), and were determined by visual inspection and palpation of the muscle (Botter et al. 2011). Starting at the lowest NMES-level that was possible to set on the NMES-device (corresponding to 4 mA), the MP scan pen was used to search through one whole side of the posterior calf in accordance with the NMES-device instruction, during which time the examiner visually checked for any sign of a muscle twitch of the calf. If a visual muscle twitch was detected, the MP pen was held still in the location inducing the muscle twitch, and the muscle twitch was via manual palpation either confirmed as true or false. If true, the location on the skin underlying the tip of the MP scan pen was confirmed as a MP point (Gobbo et al. 2014). If no MP was found, the current intensity was increased by one NMES-level followed by a subsequent re-scan. This procedure was then repeated until a visible muscle twitch was detected in the calf, indicating the location of a MP, which was subsequently marked out on the scanned side of the calf (medial or lateral). All MP scans were performed by the same person to ensure that there was no examiner-bias.

### NMES-Settings

For both electrode setups, NMES was applied using the same NMES device as for the MP scan. The NMES stimulation used a biphasic symmetric square wave, meaning that the electrodes continuously were switching polarity so that they alternately, and for equally long durations, served as either anode or cathode. Thus, there was no designated anode or cathode in the electrode setups for this study. Based on previous studies on NMES discomfort, stimulation settings where set to 36 Hz frequency, 200 µs phase duration (400 µs pulse duration), 0.5 s ramp up time and 0.25 s ramp down time (Baker et al. 1993). The duration of each stimulation between the ramp up and ramp down time, i.e., the plateau time, was varied for different tests between 0.5 s, 1.5 s, 3 s and 5 s. The muscle rest between each cycle of combined ramp up, plateau time and ramp down, i.e., the OFF-time, was 8 s. The order in which the plateau times were tested was randomized. The NMES-level (0–999), representing a non-linear relationship to the current intensity ranging from 0 to 120 milliampere (mA), was gradually increased one NMES-level at a time as described below.

### NMES Measurement Level I & II

The NMES-device display the current used for the selected stimulation in NMES-levels ranging from 0 to 999, which in a non-linear pulse duration dependent fashion correlate to current amplitudes ranging from 0 to 120 mA. The formula to calculate this correlation may be obtained from the manufacturer (DJO Nordic, Malmoe, Sweden) upon reasonable request, but may not be publicly distributed.

When testing the two electrode setups, outcomes were registered at two distinct current intensities, designated *measurement level I* (ML I) and *measurement level II* (ML II). For every test performed, the current intensity was slowly increased one NMES-level at a time until a visible plantar flexion was induced. The current intensity needed to induce this plantar flexion was defined as ML I. A clearly visible plantar flexion was chosen as a point for outcome measure because, (1) it can be dichotomised; either you can see a plantar flexion, or you cannot, and (2) it will likely increase PVV compared to the baseline resting state (Clarke et al. 2006; Laverick et al. 1990). To avoid any examiner bias, only one examiner was used for all participants for the assessment of when a visible plantar flexion was induced by the NMES. ML II was defined as the current intensity corresponding to ML I plus an additional six NMES-levels increase on the NMES-device. For example, if plantar flexion was induced at NMES-level 20 (ML I), ML II would correspond to NMES-level 26 (= 20 + 6). The six-level NMES increase was chosen based on pre-testing indicating a significant increase of PVV compared to ML I, while any further intensity increases indicated to result in significantly more aborted test due to discomfort.

Since ML I and ML II could occur at different NMES-levels for different participants, correlating non-linearly to different mA amplitudes for different participants, we deemed the presentation of the testing procedure in mA amplitudes to be too unpractical to be easily reproducible in a clinical setting, since it would require the user to obtain the conversion formula and convert the mA amplitudes back into NMES-levels. Thus, only the NMES-levels are used to describe the different current intensities during the testing procedure, while the current intensities in the results are presented in mA for better relative comparisons in the statistical analysis. The range of increase in current between ML I and ML II is disclosed in the results section.

### Hemodynamic measurements

Using a Philips CX50 (2013) Doppler ultrasound machine (Philips Medical Systems, Andover, MA, USA), the widest accessible part of the popliteal and femoral veins was located and visualized in a longitudinal plane before beginning the hemodynamic measurements. The diameters of the veins were calculated at baseline. The diameter of the popliteal vein was 0.90 ± 0.29 cm and of the femoral vein 1.05 ± 0.26 cm. For the two electrode setups (TTE & MPE), PVV was measured in the popliteal and femoral veins at baseline (i.e., electrodes attached but no NMES administered), at ML I, and at ML II. For each subject in the study, all hemodynamic measurements were performed by the same ultrasonographist. During three consecutive NMES-stimulation cycles venous measurements were recorded, and subsequently the peak venous velocity (PVV) was assessed. The measuring tool on the ultrasound machine provided, when using doppler, the ability to save the recordings of blood flow in cm/s and measure the peak venous velocity after the stimulation was complete. The diameter of the vein during the stimulation was not calculated. PVV measurements during three NMES-stimulation cycles were performed and the mean of the three was used for statistical analysis. After analyzing the refill time of the veins, an eight second OFF-time was decided to be used between ON-times for the veins to be adequately refilled with blood before the next upcoming muscle contraction and PVV measurement. To quantify the potential benefit of NMES versus the baseline resting state, for each subject and setting, the percentual increase in PVV at ML I and ML II*,* as compared to PVV at baseline, was calculated and presented along with the absolute values. The formula used to calculate the percentual increase was:

$$\left( {\text{Percentual increase}} \right) \, = \, \left( {\left( {\left( {\text{PVV with stimulation}} \right) {-} \left( {\text{PVV at baseline}} \right)} \right) / \left( {\text{PVV at baseline}} \right)} \right) \times 100$$

### Discomfort

For each stepwise increase in NMES-levels when testing the two electrode setups, participants were asked to fill in a form to rate their discomfort on a numerical rating scale (NRS) 0–10, where 0 was described to the subject as no discomfort and 10 as the worst imaginable discomfort (Hawker et al. 2011).

### Statistical analysis

The sample size was determined prior to the start of the experiment based on a pilot study with a difference in PPV between ML I to ML II of 20 cm/s and sigma of 20, with the significance level set at *p* < 0.05 and power at 80% regarding the primary outcome, PVV in the popliteal vein. Based on the calculations, eight participants were needed to find a significant increase in PVV with an increase of the current intensity from ML I to ML II. We set the final sample size to ten participants.

The data were analyzed using SPSS version 27 (IBM Corp. Released 2016. IBM SPSS Statistics for Windows, Armonk, NY: IBM Corp.) in cooperation with a statistician. The four tested plateau times, 0.5, 1.5, 3 and 5. seconds, did not demonstrate any statistically significant difference regarding PVV or NRS, regardless of the electrode setup or measurement level. For this reason, the outcome values for PVV and NRS used in the final statistical analysis were based on all values, for all participants, obtained during the four different plateau times. Based on the relatively small number of participants and the Shapiro–Wilk indicating non normal distribution, the non-parametric Wilcoxon signed rank test was chosen to determine if there were any statistically significant differences between medians regarding current intensity (mA), PVV or NRS, for the two electrode setups. The data included some outliers, which were handled both using a rank-based statistics test and by adjusting the values of the outliers to the lowest/highest value within 1.5 times the interquartile range from the first respectively third quartile for the inferential statistics (Altman 1990). Analysis of correlations between subject characteristics and collected data were performed using Spearman’s *ρ*. Data are presented with median, interquartile range (IQR) and when comparisons were made the significance level for all analyses was set to *p* < 0.05. For the remainder of the text, “statistically significant” will be shortened as “significant”.

## Results

### Current intensities in mA for calf-NMES

**Fig. 3:**
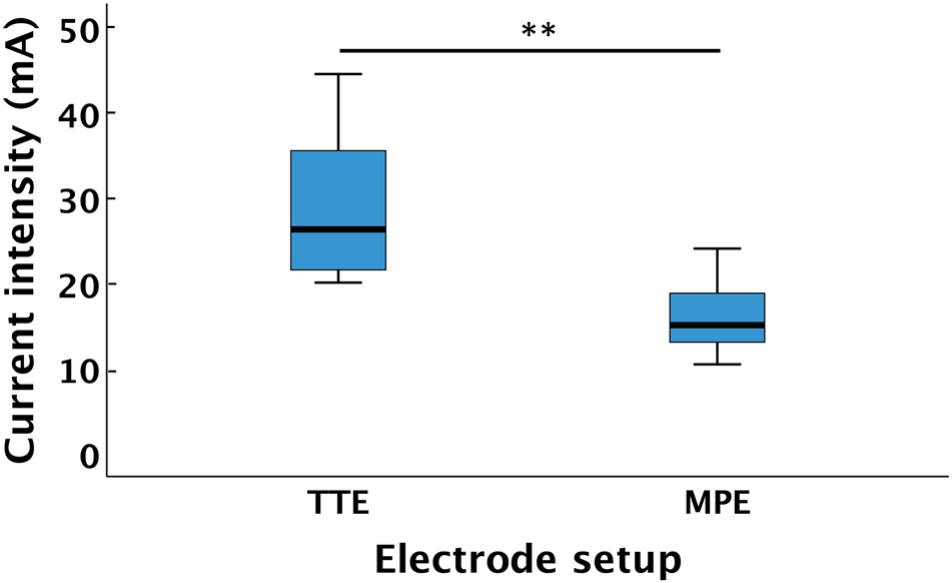
Current intensity (mA) required to reach ML I displayed for the TTE and MPE setups. In the boxplot, the length of the box represents IQR, and error bars represent min–max. The black line within boxes represents median. **Indicates a difference with *p* < 0.001. *mA*  milliAmpere. *ML*  measurement level, *TTE*  transverse textile electrode, *MPE*  motor point electrode

The median (IQR) current intensity required to reach ML I, was significantly lower when using MPE, 16 (7.4) mA, as compared to TTE, 26 (14) mA (*p* = 0.005). To reach ML II*,* the MPE setup also required a significantly lower current intensity, 21 (5.8) mA, compared to the TTE setup, 29.5 (13) mA (*p* = 0.005) (Fig. 3). The mean increase of current between ML I and ML II was 4 mA and ranged between 1.5 and 6.5 mA.

### Hemodynamics of calf-NMES

#### Hemodynamics in the popliteal vein

**Fig. 4:**
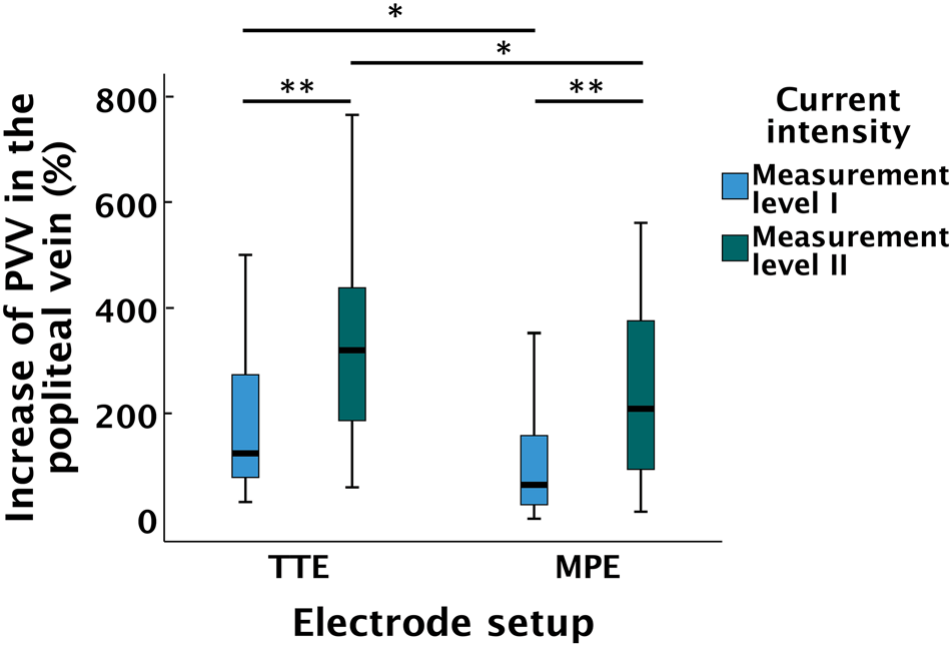
Hemodynamics in the popliteal vein. Percentual increase of PVV from baseline to ML I and ML II displayed for the TTE and MPE setups. In the boxplot, the length of the box represents IQR, and error bars represent min–max. The black line within boxes represents median. *Indicates a difference with *p* < 0.05 and **Indicates a difference with *p* < 0.001. PVV = *peak venous velocity*. *ML*  measurement level, *TTE*  transverse textile electrode, *MPE*  motor point electrode

The median (IQR) baseline PVV in the popliteal vein was 14.3 (5.4) cm/s. There were significant increases of PVV from baseline to ML I when using both TTE, 29.1 (47.3) cm/s (*p* < 0.001), and MPE, 21.7 (15.3) cm/s (*p* < 0.001) (Fig. 4). The TTE setup resulted in a significantly higher percentual increase of PVV from baseline to ML I in the popliteal vein compared to the MPE setup (*p* = 0.005).

Increasing the current intensity to ML II resulted in significantly higher increases of PVV from baseline as compared to ML I, for both the TTE- and the MPE setups (both *p* < 0.001) (Fig. 4). The PVV at ML II was 62.0 (53.4) cm/s when using TTE and 38.6 (34.7) cm/s when using MPE. The TTE- versus MPE setup resulted in a significantly higher percentual increase of PVV from baseline to ML II (*p* = 0.026) (Fig. 4).

### Hemodynamics in the femoral vein

**Fig. 5:**
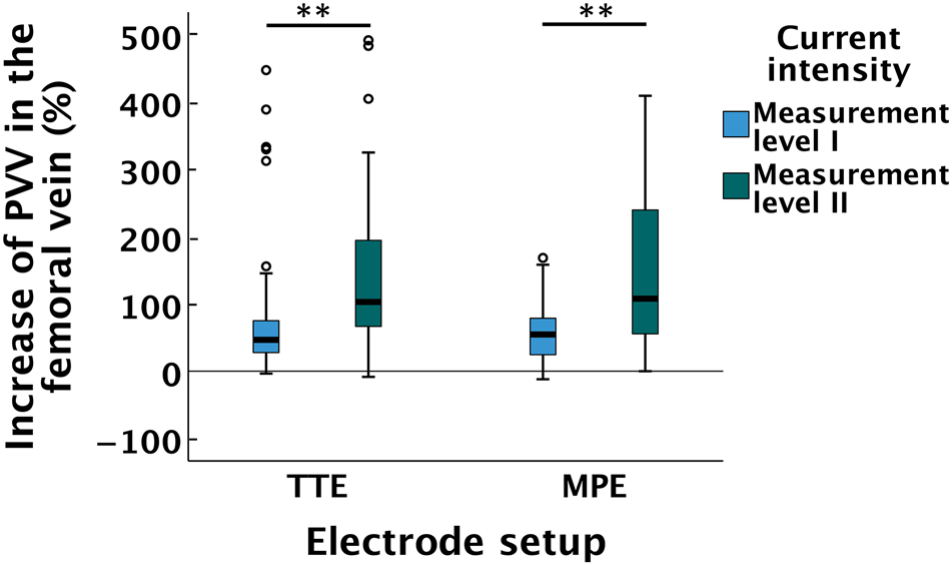
Hemodynamics in the femoral vein. Percentual increase of PVV from baseline to ML I and ML II for the TTE and MPE setups. In the boxplot, the length of the box represents IQR, and error bars represent min–max and in case of outliers 1.5xIQR. Circles represents outliers. The black line within boxes represents median. **Indicates a difference with *p* < 0.001. *PVV*  peak venous velocity, *ML*  measurement level, *TTE*  transverse textile electrode, *MPE*  motor point electrode

The median (IQR) baseline PVV in the femoral vein was 14.2 (3.9) cm/s. PVV exhibited statistically significant increases from baseline to ML I when using both TTE, 21.6 (8.8) cm/s (*p* < 0.001), and MPE, 19.2 (7.8) cm/s (*p* < 0.001) (Fig. 5). The percentual increase of PVV from baseline to ML I when comparing the TTE and MPE setups did not differ significantly (*p* = 0.635).

The increase in current intensity to ML II caused significantly higher increases of PVV from baseline as compared to ML I, when using both TTE and MPE (both *p* < 0.001) (Fig. 5). The PVV at ML II was 33.3 (20.9) cm/s when using TTE and 27.8 (26.2) cm/s when using MPE. There was no significant difference between TTE and MPE in the increase of PVV from baseline to ML II (*p* = 0.700).

### Discomfort of calf NMES

**Fig. 6:**
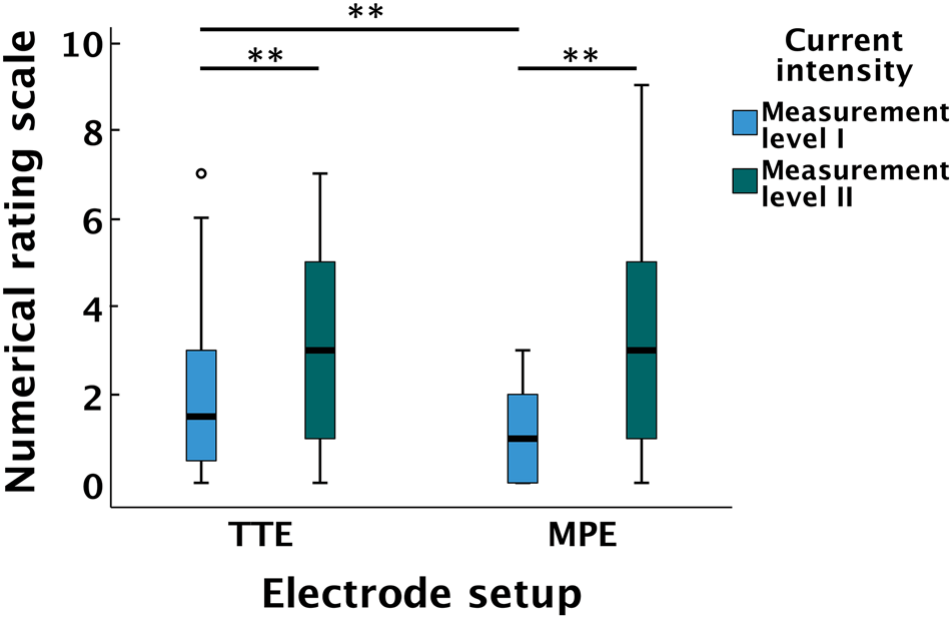
Discomfort of calf NMES. NRS score for the TTE and MPE setups at ML I and ML II. In the boxplot, the length of the box represents IQR, and error bars represent min–max and in case of outliers 1.5 × IQR. Circles represents outliers. The black line within boxes represents median. **Indicates a difference with *p* < 0.001. *NMES * neuromuscular electrical stimulation. *NRS*  numerical rating scale, *TTE*  transverse textile electrode, *MPE*  motor point electrode

Using MPE resulted in a statistically significantly lower median NRS (range), 1 (0–3), compared to the use of TTE, NRS 2 (0–7), at ML I (*p* < 0.001). At ML II, the two electrode setups did not demonstrate any significant difference in NRS (*p* = 0.836). However, at ML II, participants reported significantly higher NRS with both types of electrode setups compared to ML I (*p* < 0.001) (Fig. 6).

### Associations of participant characteristics with outcome

Female participants, as compared to male participants, required significantly higher current intensity (mA) to produce a plantar flexion, both with TTE and MPE (*p* = 0.005, *p* = 0.001). Females were also shown to have a higher percentage of body fat (*p* = 0.014). None of the participant characteristics including age, gender, BMI, body fat percentage or waist circumference were significantly correlated with NRS or the percentual increase of PVV in the popliteal or femoral veins.

## Discussion

The main finding of this study was that the PVV in both the popliteal and the femoral veins exhibited a dose–response relationship to the current intensity administered to the calf, for both the TTE and the MPE setups. The TTE setup required higher current intensity than the MPE setup to induce a plantar flexion (ML I) and to reach ML II, but also demonstrated superior increases of PVV in the popliteal vein at both ML I and ML II. However, PVV in the femoral vein was equally increased with both setups. Moreover, at ML I, the MPE setup caused less discomfort than the TTE setup. None of the participant characteristics caused more discomfort or significantly altered the increase of PVV.

The finding that the PVV in both the popliteal and the femoral vein exhibited significant median increases of 50–100% from baseline to ML I conform with results from earlier studies on NMES (Williams et al. 2017; Praxitelous 2018). However, to the best of our knowledge this is the first study demonstrating improved venous hemodynamic effects of reusable textile electrodes transversally knitted into a sock, which provides a pre-defined placement of the electrodes based on a *motor point map* (Botter et al. 2011). Moreover, the observation that these venous flows could be enhanced an additional 2–3-times, both with MPE and TTE, by increasing the current intensity of the NMES by a mean of 4 mA, suggests an intensity-dependent response between the applied current intensity and the PVV produced.

The finding of an intensity-dependent relationship between the current intensity of applied NMES (frequency: 36 Hz) and increased venous return is supported by the literature (Corley et al. 2009), but novel when it comes to textile electrodes as well as assessments of blood flow in the popliteal and femoral vein in the same individual. An earlier study strengthens our finding by demonstrating a relationship between the current intensity administered via NMES (frequencies: 1, 3 and 5 Hz) and increased venous velocity in the femoral vein (Tucker et al. 2019). Moreover, another study corroborates our observations in the popliteal vein by demonstrating that different participants, who used NMES with various current intensities (frequency: 36 Hz) exhibited PVV that on a group level were dependent on the current intensity (Corley et al. 2009).

The presumable explanation to the observed dependence between current intensity and increased PVV lies in the earlier knowledge that there is a relationship between the current intensity and muscle force production (Flodin et al. 2022; Glaviano et al. 2016). In general, a higher current intensity will cause more muscle fibers to contract (Doucet et al. 2012), which in turn increases the squeezing of the calf veins, filled with approximately 100–150 ml blood (Christopoulos et al. 1987), subsequently inducing enhanced PVV (Tucker et al. 2019). This, however, is only true for a certain range of current intensity, as there is both a lower and upper limit to which the muscle torque is correlating to changes in current intensity.

An intensity-dependent increase in venous blood flow velocity using calf-NMES is important for the prevention of DVT and VTE, since it may reduce one of their underlying causes, i.e., venous stasis in the lower limbs (Hajibandeh et al. 2015; Williams et al. 2017). The minimum hemodynamic effect of NMES required to prevent development of DVT is unknown. Comparable data for hemodynamics during voluntary activation of the calf muscle pump, however demonstrated at least a twofold increase in PVV (Clarke et al. 2006; Tsuda et al. 2020; Toya et al. 2016). Thus, our observed two–threefold increases in PVV in the popliteal and femoral veins may reflect clinically relevant enhancements of venous velocity for preventing DVT. This effect would be especially beneficial if it could be integrated into garments that are used every day, such as socks, since it likely would render the compliance to treatment greatly improved.

The second most important finding of this study was the identification that the TTE setup, produced an equal or even better hemodynamic response in the popliteal vein compared to the MPE setup. The most plausible explanation to the enhanced hemodynamic response seen would be that the TTE setup required a higher current to reach ML I. This may theoretically have resulted in a broader stimulation over the muscle bulk, activating more muscle fibers as compared to the more focused stimulation resulting from the MPE setup. The TTE setup was configured with electrodes transversely, approximately at the thickest portions of both the lateral and medial gastrocnemius muscle heads, which may be a good electrode placement to cause a broader muscle fiber activation for better venous return (Uhl et al. 2015).

However, since the determination of when a plantar flexion was performed did not differ between the two electrode setups, another explanation to the observed increased hemodynamic response with TTE may be that the higher current intensity also induced indirect, local effects on blood vessels via the nervous system (Maffiuletti et al. 2018). It has been demonstrated that electrical stimulation causes sympathetic nerve fiber activation, which may locally constrict the popliteal vein with subsequent increase in blood flow velocity (Stefanou et al. 2016). The latter explanation may further be supported by the finding that the TTE- vs the MPE setup produced a better hemodynamic response in the popliteal vein, but not in the femoral vein, suggesting that the higher electrical current administered over the calf induced local constriction effects over the popliteal vein, but not over the femoral vein which was further away from the source of the electrical stimulation. Further studies, however, should explore how different electrode placements on the calf may optimize the effect of the muscle-vein pump and whether these effects are related to direct intensity-dependent muscular contraction or related to indirect activation of the nervous system.

The observation in this study of a more pronounced response of the calf-NMES treatment in the popliteal vein as compared to the femoral vein could most reasonably be explained by two factors. The distance from the calf to the popliteal vein is shorter than to the femoral vein and the diameter of the popliteal vein is smaller as compared to the femoral vein. Thus, changes in peak venous velocity after calf-NMES treatment will be more easily detected in the popliteal as compared to the femoral vein. Another possible influencing factor may be the body position. However, in this study patients were analyzed in the semirecumbent position with the legs horizontal, mimicking a clinical situation in a hospital bed. Thus, the observed increases in venous velocity in the femoral vein may be of importance for patient with eg. Hip- and pelvic fractures, in which blood clots may develop in more proximal veins. However, this conclusion will necessitate further studies.

Another aspect to consider when deciding the optimal placement of electrodes is the discomfort of the patients. The observation that the MPE setup exhibited less discomfort at ML I as compared to the TTE setup suggests a better compliance with treatment when electrodes are placed on MP, which is in line with earlier studies (Gobbo 2011). The finding that there was a difference in hemodynamic effect, but no difference in discomfort between the MPE and TTE setup at ML II, on the other hand, suggests that a compromise of several aspects must be taken into account to optimize patient outcome. For example, that compliance to treatment is also affected by the usability of the NMES electrodes. A standardized sock does not require the motor point scan and should therefore increase the usability. However, the issue with discomfort needs to be solved.

The great differences in PVV observed, both at baseline and with calf-NMES, are suggestive of vast individual variations, which are supported by earlier studies (Evans et al. 2016). This study also investigated the participant characteristics in relation to the outcome variables. The observation that females required higher current to obtain a plantar flexion of the ankle suggests that differences in subcutaneous fat composition may impede the flow of the electrical current, as has been suggested before (Maffiuletti 2010). The differences between sexes seen in electrical current required to reach ML I, did however not cause dissimilarities in discomfort or hemodynamics, suggesting that both sexes may benefit equally from calf-NMES. However, given the relatively small number of participants in this study conclusions regarding subgroups are difficult to make with certainty. Participant characteristics did in this study not explain the great interindividual PVV variation in response to stimulation. To explore factors related to potential non-responders would be of interest for further research.

A possible limitation of the study is the relatively low number of participants, which hinders conclusions especially on associations between subject characteristics and outcome. Venous velocity, which was assessed with Doppler ultrasound, can be influenced by a number of factors such as venous pressure and vein diameter that were not assessed during stimulation. However, the vein diameter was assessed at baseline and since the stimulation time was so short it is less likely that it affected the blood pressure or the release of cortisol to change the vein diameter. Another limitation is the potentially differing impedances of the TTE and MPE setups, as well as of the skin of the different participants, which is likely to have influenced results. However, we believe that such measurements would have been unreliable considering that manipulation of the calf in the form of ultrasound measurements and NMES, among other factors (Euler et al. 2021a, b). The main purpose of this study was not primarily to compare isolated factors related to NMES, but rather to compare two different concepts in the form of two different electrode setups, which may represent future alternatives for NMES users.

## Conclusion

NMES of the calf increases PVV in an intensity-dependent and clinically relevant manner in both the popliteal and femoral veins using both a TTE*-* and a MPE setup. The MPE setup requires a lower mA amplitude which seem to reduce discomfort during NMES. The TTE setup, however, seems to enhance PVV in the popliteal vein to a higher extent, plausibly due to higher mA amplitude required, as compared to the MPE setup. Textile electrodes are promising for enhancement of venous hemodynamics, potentially increasing compliance due to easy of use and offering a reusable alternative to standard electrodes. However, there is a need of further development in reducing the discomfort of textile electrodes to improve patient compliance.

## Data Availability

The datasets used and/or analyzed during the current study are available from the corresponding author on reasonable request.

## Abbreviations

DVT: Deep vein thrombosis
MP: Motor point
MPE: Motor point electrode
NMES: Neuromuscular electrical stimulation
NRS: Numerical rating scale
PVV: Peak venous velocity
TTE: Transverse textile electrode
VTE: Venous thromboembolism
